# Letter from Professor J. A. Eve to the Editor of the Medical and Surgical Journal, on the Use of Chloroform in Obstetric Practice

**Published:** 1848

**Authors:** J. A. Eve


### ARTICLE II.

*Letter from Professor* J. A. Eve *to the Editor,* [*of the Southern Medical and Surgical Journal,*] *on the use of Chloroform in Obstetric Practice.*

March 15th, 1845.

Dear Doctor—Before taking leave of our last class, I expressed to them my thorough conviction of the safety and utility of the Chloroform in Obstetric Practice; and these have certainly been satisfactorily demonstrated by Profesor Simpson, to whom the gratitude of the gentler sex and the medical profession is due for having introduced it into obstetrics. And its safety and utility determine the propriety; for it is absurd to object to its use as an attempt to countervail the Divine decree which said to our first mother "in sorrow thou shalt bring forth." The argument would be equally good against all obstetric assistance, and indeed of equal force against any profession or calling in life, by which any descendant of Adam may "eat bread," otherwise than "in the sweat of his brow." It is certainly much more consonant with sound reason and true religion, to regard all the discoveries and improvements in Science and Art as the blessings of God, given to soften the severity of the primeval curse, and meliorate man's fallen state, without respect to person or sex.

But whilst the safety of chloroform, in properly selected cases, has been demonstrated, it is unreasonable to suppose that an agent, so powerful in its action on the nervous system as entirely to suspend sensibility, can be altogether devoid of danger, in some cases, and no caution requisite in its administration.

My opinion is, that it should only be administered in labors of unusual severity, or wherein some capital obstetric operation is required, and never, unless the system is free from disease, especially from those of the head, or even a predisposition thereto. That it should rarely, if ever, be given before the termination of the first stage; that the patient should not be kept under its influence more than one or two continuous hours, but be allowed to wake up, and after a short interval be put to sleep again, as often as may be considered necessary.

On the 4th instant, I administered the chloroform to a lady whose former labors had been very painful and protracted: at a quarter past 10 o'clock, P. M., soon after the second stage had commenced she began to inhale it simply poured on a handkerchief, as a piece of sponge could not be procured. She soon fell asleep, but the uterine contractions continued with equal force and more effect. My first remark was, that the contractions were more efficient in advancing the labor, though further apart, but on timing the intervals by a watch, it was found that they were only apparently long, from the intense anxiety with which I waited for the uterus to act, and that sometimes the interval was not more than two or three minutes. Her pulse became slower. Her respiration was sometimes audible, but never stertorous; during most of the time she slept and breathed as sweetly and softly as an infant. She would occasionally wake up partially, and eagerly grasp and press the handkerchief to her nose to deepen the effect. She continued under the influence of the chloroform an hour and a quarter, unconscious of pain until after the expulsion of the head, when she awoke and earnestly entreated for more, but the supply was exhausted. She declared that if she had had more, the whole birth would have been accomplished without her knowledge; but even as it was, she said she did not suffer near as much, as she otherwise would have done, from the last pain or two required to terminate the delivery, in consequence of having had so long a respite from pain.

I confidently believe, had she not taken the chloroform, her labor would have continued some hours longer, as on former occasions, when her children were much smaller than in this instance; the child weighed eleven pounds.

The agency of chloroform in accelerating labour is most probably attributable, in a great degree, if not principally, to its effect in rendering the patient silent and motionless, when no power being expended in exclamation and motion, all the energies of the system are concentrated on the uterus.

This lady compared the sensation excited in her head, by chloroform, to the sound of ten thousand hammers, but it was not at all painful or even unpleasant. She expressed herself in the highest degree delighted with its effects, and thanked Heaven that she had had resolution enough to take it—that hereafter she would not be afraid to use it for any purpose.

Not the slightest injury was sustained, either by mother or offspring.

Since the above was written, there has been published in the Medical Examiner a letter from Professor Meigs to Professor Simpson, in which the former gentleman objects to chloroform, in obstetric practice—1st, because he regards the pain in parturition as physiological, and thinks it ought not to be suppressed, and 2dly, because in the employment of instruments, he considers it necessary to know how much the patient suffers to avoid doing harm.

The first objection appears to me rather more specious than solid, for admitting the pain to be physiological, I can perceive no good reason why it should not be relieved, if susceptible of relief, without interference with the process or injury to the patient. But it is a difficult question to determine how much pain is strictly physiological, or essential to parturition, and how much is the result of the unnatural and injurious habits and customs of civilized life; for we know that in the savage state and among those whose manner of living is more natural and conducive to the healthy performance of all the physical functions, there is comparatively very little pain or danger; and even in the most refined society, instances are not very uncommon of almost painless labour.

In what are styled easy labors, or whenever the suffering is not intense, anæsthesia is scarcely necessary, even if there were no danger at all. But pain, when inordinate, is itself a great source of danger, as it may destroy life directly, or indirectly, by inducing convulsions or other fatal affections: it should certainly, therefore, be at least abated, whether physiological or pathological. If there were no dangers to be apprehended from pain, it is in itself a great evil, and its relief an object of vast importance, one of the happiest achievements and highest triumphs of medicine. Surgical statistics declare a diminished mortality, under the use of anæsthetic agents, and I think it highly probable there will hereafter be the same result in obstetrics.

Professor Meigs' second objection does not appear to me much more valid than the first. The degree of pain actually felt is not always in proportion to the injury sustained, and the expression is a still more fallible criterion. Some complain excessively when suffering comparatively very little, whilst others make very little complaint, however intense their sufferings may be. Nothing can compensate to the accoucheur for a want of the most minute and accurate knowledge of the maternal structures and the position and relations to them of the fætal head. He should always operate by art and not by force, graduating the power employed by his knowledge of what may be applied and borne with safety, and not by the resistance to be overcome: he should always know that he is using no destructive violence, inflicting no injury, whether the patient be sensible of it or not. It would certainly be a very uncertain and unsafe reliance, to depend on the patient to inform us whether we are applying and using instruments properly. One great source of embarrassment and danger in operative midwifery is the difficulty often encountered, of controling the voluntary movements of the patient, which is entirely obviated by the anæsthetic influence of the chloroform. It is in instrumental deliveries, and during the performance of other important obstetric operations, that I consider its administration peculiarly appropriate and advantageous.

With sincere regard, your friend,

J. A. EVE.

—*Southern Med. and Sug. Journal.*

